# Effect of the Field of View Size on CBCT Artifacts Caused by the Presence of Metal Objects in the Exomass

**DOI:** 10.1155/2022/2071108

**Published:** 2022-09-09

**Authors:** Yaser Safi, Mitra Ghazizadeh Ahsaie, Maede Jafarian Amiri

**Affiliations:** Department of Oral and Maxillofacial Radiology, School of Dentistry, Shahid Beheshti University of Medical Sciences, Tehran, Iran

## Abstract

**Materials and Methods:**

In this in vitro experimental study, titanium implants, teeth with cobalt-chromium (Co-Cr) intracanal posts, and teeth with mesio-occluso-distal (MOD) amalgam restorations were placed in an empty socket of the extracted third molar of a human mandible. These metallic materials were differently arranged in the exomass (zone outside of the FOV). A polypropylene tube containing dipotassium phosphate was placed in the empty socket of the right canine tooth in a dry human mandible. CBCT scans were taken with a NewTom VGI (Verona, Italy) scanner using a 6 × 6 cm and an 8 × 8 cm FOV. The histogram tool of OnDemand software (Cybermed, Seoul, Korea) was used to select circles with a 1.5 mm diameter as the (ROI) at the center of the homogenous solution of dipotassium phosphate tube on the axial plane. The mean gray value (GV) and its standard deviation (SD) in the region of interest (ROI) were calculated (*P* > 0.05). The data were analyzed by SPSS 26.

**Results:**

The reduction in the size of the FOV significantly decreased the mean GV (*P* < 0.001). Metal objects in the exomass significantly decreased the mean GV (*P* < 0.001), and minimum mean GV and maximum SD were recorded for amalgam, followed by Co-Cr intracanal posts, and titanium implants. The unilateral presence of a metal object was associated with a higher mean GV and lower SD (*P* < 0.001).

**Conclusion:**

Using a smaller FOV increases the size of the exomass, which may negatively affect the image quality. Metal objects in the exomass decrease the GV of CBCT scans and adversely affect the image quality.

## 1. Introduction

Dental radiography is a fundamental tool in clinical diagnosis and treatment planning and is commonly requested by dental clinicians. At present, dental implants are increasingly used for the replacement of lost teeth. However, dental implant treatment requires highly accurate imaging modalities for preoperative assessment of intraoral conditions and bone quality [1].

Cone-beam computed tomography (CBCT) is a valuable imaging modality that provides three-dimensional images of the maxillofacial structures [2]. CBCT has optimal (<1 mm) resolution and isotropic voxels. It also has a smaller size, lower cost, higher scanning speed, lower patient radiation dose, and stronger reconstruction software compared with computed tomography, and is, therefore, more practical for dental applications. CBCT can greatly enhance the diagnosis, treatment planning, and follow-up of patients in different dental fields such as implantology, oral and maxillofacial surgery, endodontics, and orthodontics [3, 4]. However, despite all the advantages and popularity of CBCT and its extensive applications in dentistry, artifacts decrease the quality of CBCT images and complicate their interpretation. Artifacts cause a difference between the reconstructed image and the actual image content [5]. Beam hardening artifacts, motion artifacts, streak artifacts, circular artifacts, and exomass-related artifacts [6] are among the different types of artifacts that compromise image quality. CBCT artifacts decrease the contrast, mask the structures, compromise image quality, and subsequently complicate correct diagnosis [2]. Beam hardening artifacts are among the most important artifacts generated in the presence of high-density structures with high atomic numbers such as titanium implants, amalgam restorations, and metal prostheses, and significantly decrease the image quality [7]. It has been well accepted that metal restorations and dental implants are the main causes of CBCT artifacts [8, 9]. The composition, number, and location of metal objects can variably affect the quality of CBCT images [10–13].

Recently, the use of a small field of view (FOV), referred to as the local tomography technique [14], has increased due to lower patient radiation dose and higher image quality [11]. In the use of small FOV, only a small part of an object is scanned, and the data obtained from the exomass, i.e., structures located out of the FOV and between the focal spot and image receptor, are eliminated to prevent negative interferences. However, it has been confirmed that the presence of metal objects in the exomass decreases the gray value (GV) and causes artifacts on reconstructed images [15, 16]. Even the metal artifact reduction (MAR) algorithms cannot decrease such artifacts [17].

Considering the extensive use of metals in dental treatments and the increasing application of small FOVs, this study aimed to assess the effect of the size of FOV on artifacts caused by metal objects in the exomass.

## 2. Materials and Methods

The study protocol was approved by the ethics committee of Shahid Beheshti University of Medical Sciences (IR.SBMU.DRC.REC.1399.132).

### 2.1. Sample Preparation

In this in vitro experimental study, CBCT scans were taken from a dry human mandible with partial edentulism.

#### 2.1.1. Exomass Preparation

To induce exomass-related metal artifacts, three types of metal objects were used:

1. Titanium dental implants with 4.5 mm width and 10 mm height (Snucone, Korea)
2. Teeth with Co-Cr intracanal posts (Neodontics,Inc.USA)
3. Teeth with mesio-occluso-distal (MOD) amalgam restorations (SDI, Victoria, Australia)

Metal objects were further placed in the dry human mandible in the following two states:

- A metal object in the empty socket of a right third molar tooth
- Two metal objects in the empty sockets of right and left third molar teeth

**Figure 1:**
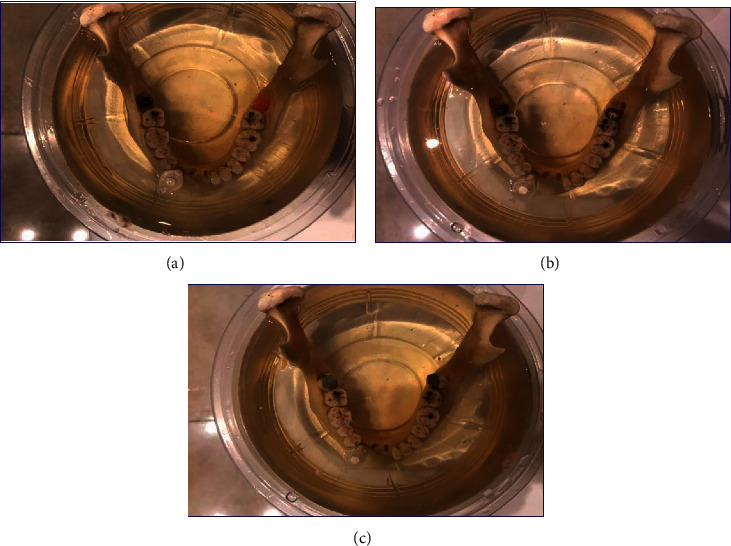
Placement of the titanium implant (a) cobalt-chromium intracanal post (b) amalgam restoration (c) and in a dry human mandible. The bodies of the phantoms were made of thin plastic and filled with ballistic gelatin.

Titanium dental implants were fixed in the empty tooth sockets with dental max. Co-Cr intracanal posts were placed in the root canals of extracted premolars, and the teeth were fixed in the empty sockets of third molars in the dry human mandible with dental wax. MOD amalgam restorations were performed for extracted premolar teeth, and they were placed in the empty sockets of third molars in the mandible (Figure 1). The bodies of the phantoms were made of thin plastic.

#### 2.1.2. Sample Preparation for Quantitative GV Assessment

A dipotassium phosphate homogenous solution (K2HPO4) was prepared at a 1000 mg/mL concentration and transferred into a polypropylene tube with a 0.2 mL volume, 15 mm height, and 5 mm diameter. This concentration was selected to simulate alveolar bone density [18]. The polypropylene tube containing dipotassium phosphate was placed in the empty socket of the right canine tooth in a dry human mandible and served as the site of ROI evaluation.

**Figure 2:**
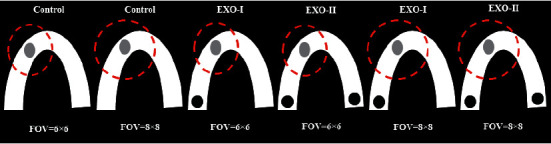
Schematic view of the mandible and position of metal objects relative to the polypropylene tube. Exo: exomass, FOV, the field of view.

After mounting metal objects in the dry human mandible, the sample was placed in a plastic container with a 15-cm diameter, and the container was completely filled with ballistic gelatin to simulate the soft tissue. Ballistic gelatin was prepared as follows according to a previous study [19]: 48 g of colorless gelatin (Sigma–Aldrich, Germany), 200 mL of glycerine (Sigma–Aldrich, Germany), and 500 mL of water were mixed and heated for 25 minutes to boil. After 20 minutes of cooling at room temperature, ballistic gelatin was poured into the container containing dry human mandibles and metal objects, and the plastic container was refrigerated for 8 hours. After each scanning, the scanned metal object was removed from the socket, and replaced with another metal object, followed by subsequent scanning.  Figure 2 shows the schematic view of the mandible and the position of metal objects relative to the polypropylene tube.

### 2.2. CBCT Examination Setup

The scans were obtained using a NewTom VGI CBCT scanner (Quantitative Radiology, Verona, Italy) with 110 kVp, 0.15 mm voxel size, and 10.73 mA for 6 × 6 cm FOV and 3.00 mA for 8 × 8 cm FOV. Prior to scanning, the scanner was warmed up by conducting two preview scans. Also, to minimize the effect of warming up of the scanner on the GV, 10-minute intervals were considered between the scans [20]. The dry human mandible was then placed in the FOV. Finally, 14 scans were obtained as follows:

1. Scanning the dry human mandible containing one titanium dental implant in the empty socket of the right third molar using 6 × 6 cm FOV
2. Scanning the dry human mandible containing two titanium dental implants placed in empty sockets of the right and left third molars using 6 × 6 cm FOV
3. Scanning the dry human mandible containing one titanium dental implant in the empty socket of the right third molar using 8 × 8 cm FOV
4. Scanning the dry human mandible containing two titanium dental implants placed in empty sockets of the right and left third molars using an 8 × 8 cm FOV
5. Scanning the dry human mandible containing one tooth with Co-Cr intracanal post in the empty socket of the right third molar using 6 × 6 cm FOV
6. Scanning the dry human mandible containing two teeth with Co-Cr intracanal posts in the empty sockets of right and left third molars using 6 × 6 cm FOV
7. Scanning the dry human mandible containing one tooth with Co-Cr intracanal post in the empty socket of the right third molar using 8 × 8 cm FOV
8. Scanning the dry human mandible containing two teeth with Co-Cr intracanal posts in the empty sockets of the right and left third molars using 8 × 8 cm FOV
9. Scanning the dry human mandible containing one tooth with a MOD amalgam restoration in the empty socket of the right third molar using 6 × 6 cm FOV
10. Scanning the dry human mandible containing two teeth with MOD amalgam restorations in the empty sockets of the right and left third molars using 6 × 6 cm FOV
11. Scanning the dry human mandible containing one tooth with a MOD amalgam restoration in the empty socket of the right third molar using 8 × 8 cm FOV
12. Scanning the dry human mandible containing two teeth with MOD amalgam restorations in the empty sockets of the right and left third molars using 8 × 8 cm FOV
13. Scanning the dry human mandible with no metal object using 6 × 6 cm FOV (control)
14. Scanning the dry human mandible with no metal object using 8 × 8 cm FOV (control)

Each control scan was taken twice and a total of 16 scans were made, and 10 regions of interest (ROIs) in each specimen were evaluated.

### 2.3. CBCT Volume Assessment

The on demand 3D application version 1.0.10 (Cybermed Inc., Seoul, Cybermed Inc., Seoul, Korea) was used to reconstruct each scan with a 0.1 mm isotropic voxel size, and the images were saved in DICOM format. The artifacts were quantified by OnDemand software. All assessments were made on 16-bit images on a Barco MDMC-12133 monitor (Kortrijk, Belgium) by a postgraduate student of oral and maxillofacial radiology. The coronal and sagittal planes were adjusted, and the measurements were made such that the vertical axis of the polypropylene tube containing dipotassium phosphate solution was perpendicular to the axial plane. The histogram tool of OnDemand software was used to select circles with a 1.5 mm diameter as the ROI in the axial plane. Measurements were started from part of the polypropylene tube that was completely embedded in the alveolar bone. Next, by the selection of a 1 mm slice thickness, measurements of each ROI were continued at 1 mm intervals from the coronal towards the apical region on 10 axial sections.

For the GV of each ROI, the related mean and standard deviation (SD) values were recorded. The mean GV was reported to assess the overall lightness/darkness of an image, and the SD value was reported to assess the variability of GVs or nonhomogeneity of the image [21].

To assess the intraexaminer reliability, 10 measurements were repeated after a 2-week interval. The intraclass correlation coefficient was calculated. The mean difference was also calculated. The limits of agreement and error range of GVs were also calculated using the following formula:

  Limits of agreement: (mean difference ± 1.96) × SD of difference.
  Error range: measurement error × critical value.
  Measurement error: (SD of differences)/critical value: 1.96.

Since, metal artifacts cause both light and a dark line, averaging the points does not provide accurate information regarding the mean GV. Thus, to report the mean GV for each of the 160 points, Adobe Photoshop software version 23.1.0.143 and Adobe Illustrator version 26.0.2.754 were used to provide an accurate estimate of GV. To define a color for each value, the black color was allocated to the smallest recorded value while the white color was allocated to the largest recorded value. Accordingly, different shades of gray were allocated to other values.

The data were analyzed using SPSS version 26. The normal distribution of data was evaluated by the Shapiro–Wilk test, which showed a normal distribution of data (*P* > 0.05). The groups were compared by three-way ANOVA (considering the presence of three independent variables of the size: FOV, type of metal object, and the number of metal objects), followed by multiple comparisons with Bonferroni correction at a 0.05 level of significance.

## 3. Results

The intraclass correlation coefficient was found to be 0.73–0.99, which indicated excellent intraexaminer reliability.

A total of 16 groups were compared (*n* = 10 in each group) considering the two sizes of FOV, four groups with respect to metal objects (three different metal objects and one control group with no metal object), and the number of metal objects (1 or 2, or unilateral and bilateral).

### 3.1. Mean GV

In Table 1 presents the descriptive findings regarding the mean GV based on the size of FOV and the type and number of metal objects. The Photoshop findings indicated that the gray shadows in scans of bilateral metal objects with a 6 × 6 cm FOV were darker than the scans of unilateral objects with an 8 × 8 cm FOV. The darkest gray shadows belonged to amalgam restorations, followed by Co-Cr intracanal posts and titanium implants.

Three-way ANOVA showed that the type of metal object (*P*=0.001), size of FOV (*P*=0.001), and the number of metal objects (*P*=0.044) had significant effects on the mean GV (indicating the amount of artifacts). Also, the interaction effects of the size of FOV and type of metal object (*P*=0.001), type of metal object and number of metal objects (*P*=0.001), and size of FOV, type of metal object, and number of metal objects (*P*=0.001) were significant on the mean GV. Regarding the magnitude of effect, the type of metal object had the maximum effect on the mean GV, followed by the size of FOV,and then, the number of metal objects. Of the different interaction effects, the interaction effect of type of metal object and number of metal objects was the greatest, followed by the interaction effect of size of FOV and type of metal object.

### 3.2. Type of Metal Object

**Figure 3:**
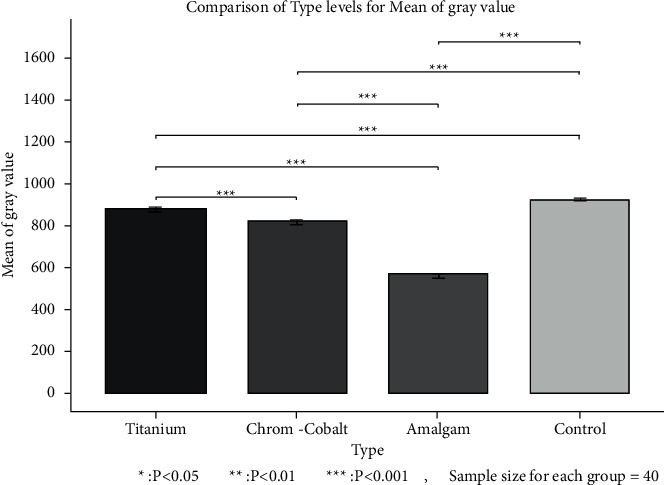
Pairwise comparisons of different metal objects regarding their effect on the mean GV.

Pairwise comparisons of different types of metal objects regarding their effect on the mean GV by the Bonferroni correction revealed significant differences between all metal objects (Figure 3). The maximum mean GV was recorded in the control group while the minimum value belonged to amalgam restoration (*P* < 0.05).

### 3.3. Size of FOV

As mentioned earlier, a significant difference existed in the mean GV regarding the size of the FOV, and the mean GV was significantly higher in the use of an 8 × 8 cm FOV (*P* < 0.001).

### 3.4. Number of Metal Objects

As mentioned earlier, a significant difference existed in the mean GV regarding the number of metal objects, and the mean GV was significantly higher in the presence of one metal object (*P* < 0.05).

### 3.5. SD of GV

In Table 2 presents the descriptive findings regarding the SD of GV based on the size of FOV and the type and number of metal objects. Three-way ANOVA showed significant effects of type of metal object (*P*=0.001), a number of metal objects (*P*=0.001), and size of FOV (*P*=0.001) on SD of GV. All interaction effects were significant as well (*P*=0.001 for all). Regarding the magnitude of effect, the type of metal object, followed by the number of metal objects, had the greatest effect on the SD of GV. Regarding the interaction effects, the interaction effect of type and number of metal objects had the greatest effect, followed by the interaction effect of all three variables.

### 3.6. Type of Metal Object

**Figure 4:**
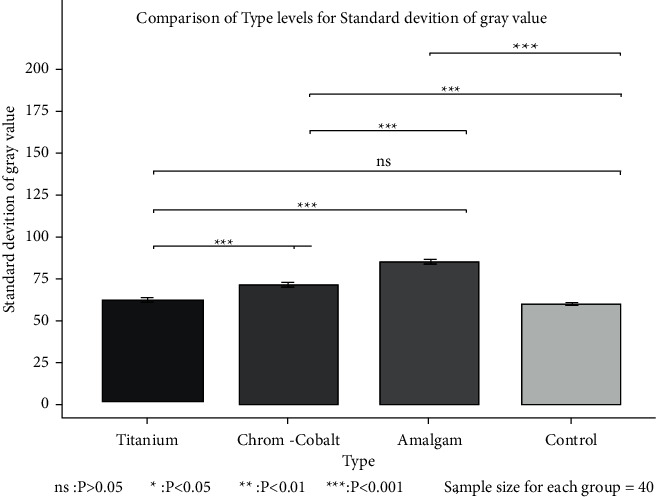
Pairwise comparisons of different metal objects regarding their effect on the SD of GV.

Multiple comparisons by the Bonferroni correction (Figure 4) revealed significant differences between all three metal objects regarding their effect on SD of GV (*P*=0.001 for all) except for the comparison of control and titanium dental implant groups (*P*=1.00). As shown, amalgam restorations yielded a maximum SD of GV from significant differences with all other groups, while the minimum SD of GV belonged to both titanium implants and the control group (with no significant difference between them).

### 3.7. Size of FOV

As mentioned earlier, a significant difference existed in the SD of GV regarding the size of FOV, and the SD of GV was significantly higher in the use of an 8 × 8 cm FOV (*P* < 0.001).

### 3.8. Number of Metal Objects

As mentioned earlier, a significant difference existed in the SD of GV regarding the number of metal objects, and the SD of GV was significantly higher in the presence of two metal objects (*P* < 0.001).

## 4. Discussion

Several strategies with variable levels of success have been proposed to decrease CBCT artifacts such as using a small FOV, using antiscatter grids, and adjustment of voltage (kVp) and amperage (mA) [22, 23]. The use of the MAR algorithm is another suggested strategy for this purpose [23]. However, none of the abovementioned strategies has been successful in the complete elimination of metal artifacts. The effects of the type and location of artifact-generating objects in the FOV and the CBCT exposure parameters on artifact generation have been previously investigated [24, 25]. However, less attention has been paid to exomass-related artifacts. This study was among the first to assess the effect of the size of FOV on CBCT artifacts caused by metal objects in the exomass. A dry human mandible was used in this study to better simulate the clinical setting.

Despite the available literature on CBCT metal artifacts, no consensus has been reached regarding an accurate method to quantify the effect of artifacts on image quality [26]. Qualitative assessments by the observers can be effective for the evaluation of artifact reduction and for diagnostic purposes. However, such subjective assessments cannot be reliably used for comparison of different protocols or for quality control purposes. There is no standard parameter to quantify the effects of metal objects on voxel values [27]. The available literature mainly measured the metal artifacts by using different parameters, such as the mean and SD of GV [6]. In areas with low GV due to beam hardening artifacts, increased mean GV along with decreased SD can indicate a reduction in metal artifacts. The mean gray shadows can provide an overall estimate regarding the level of darkness/lightness due to metal artifacts. Higher SD values can indicate higher noise and lower image quality [22]. Thus, this study assessed the mean and SD of GV. The present results showed significant effects of all three variables, namely, the size of FOV, type of metal object, and the number of metal objects, on the GV (amount of artifacts).

The size of the CBCT FOV is an important parameter in calculating patient radiation dose [16, 28]. Thus, using the smallest FOV in CBCT has been extensively recommended to minimize the patient's radiation dose [29, 30]. Under similar exposure conditions, a small FOV collimates the X-ray beams and decreases the scattered radiation, which subsequently results in a lower patient radiation dose and improved image quality [11, 31]. However, the local tomography technique indirectly increases the effect of exomass and the occurrence of the truncation effects by decreasing the size of FOV in the axial plane [14, 15, 31], and causes inconsistencies in the reconstructed volume [32]. Objects present in the exomass are radiographed but not reconstructed and change the GV as such [14]. The beam hardening artifacts caused by metal objects located outside of the FOV affect the voxel values [33]. The present results showed that decreasing the size of the FOV from 8 × t 8 cm to 6 × 6 cm decreased the mean GV, increased the artifacts, and decreased the image quality. In other words, structures in the exomass significantly affected the image quality.

Candemil et al. [16] showed that smaller FOV increased the effect of exomass-related artifacts, which was in line with the present findings and can be explained by the truncation effect. A previous study showed that small FOV, depending on the presence/absence, location, and the number of metal objects in the exomass, can negatively affect image quality [10], which is in agreement with the present findings. Kocasarac et al. [34] indicated that dental implants present in the exomass created images with higher SD and higher artifacts compared with implants within the FOV, which was in agreement with the present findings. Katsumata et al. [28] evaluated the correlation of GV and CBCT volumes using CBCT Alphard, which can provide different imaging volumes. They demonstrated that larger imaging volume due to larger voxel size was associated with lower image quality and loss of details.

Thus, according to the results of the present study, the size of FOV can significantly affect image quality, especially in presence of high-density objects. Previous studies also showed that increasing the size of the FOV decreased the variability in GV [11, 28, 35]. Under similar exposure conditions, the use of a larger FOV only to improve image quality is not justified because it increases the patient radiation dose. Thus, priority should be given to minimizing exomass-related artifacts [16]. Oliveira et al. [36] indicated that exomass-related artifacts decreased the variability of GV, which was different from the present findings. In their in vitro experimental study, exomass was simulated by using a homogenous thin layer of water, which may serve as an X-ray filter and increase the mean energy of the X-ray beam and may also be less susceptible to changes in the size of FOV.

In the present study, metal objects in the exomass created hypodense and hyperdense streaks, which are in fact due to beam hardening and scattering artifacts [22, 27]. In this study, the presence of metal objects in the exomass, irrespective of their type, decreased the mean GV of images taken by the NewTom VGi CBCT scanner. This finding indicates the dominance of hypodense artifacts on the images, which can probably have a negative effect on the detection of hypodense conditions such as fracture lines in the clinical setting. However, a previous study showed that although the presence of high-density materials in the exomass negatively affected the CBCT image quality, they had no adverse effect on the diagnostic accuracy of CBCT for detection of vertical root fractures [37]. This difference is probably due to the use of different types of scanners and optimized exposure settings applied in their study. Candemil et al. [16] demonstrated that the mean voxel value decreased in the presence of any metal object in the exomass in the use of CS9300 and Picasso Trio CBCT scanners, and the presence of two Co-Cr or amalgam cylinders in the use of the NewTom Giano scanner, which was in agreement with the present findings. However, unlike the present study, they showed that the presence of one to three Co-Cr cylinders or amalgam restorations resulted in the generation of greater amounts of hyperdense artifacts on images taken by the NewTom Giano scanner. Such a controversy in the results of different CBCT scanners is probably due to some small variations in exposure parameters such as amperage (mAs). Also, it may indicate that the artifacts generated by objects with different linear attenuation coefficients are not expressed similarly by different CBCT scanners [21].

The physical properties of objects directly affect the CBCT artifacts [16]. In the present study, the lowest mean GV and maximum SD of GV belonged to amalgam restorations, Co-Cr intracanal posts, and titanium implants. This finding may be due to differences in the atomic numbers of these metal objects. The atomic number of titanium implants is 22, while that of chromium and cobalt is 24 and 27, respectively. The atomic numbers of mercury, silver, tin, and copper in the amalgam alloy are 80, 47, 50, and 29, respectively [16, 17]. Thus, high atomic number and high physical density increase artifact generation when exposed to X-ray radiation [6, 29], and this increase is directly proportionate to the change in GV. Increased variability in GV indicates greater effects of artifacts on CBCT images, and subsequent reduction of image quality [27, 37].

In the present study, the mean GV in unilaterally placed titanium implants scanned with a 6 × 6 cm FOV was slightly lower than that of Co-Cr intracanal post. Although the Co-Cr intracanal post has a higher atomic number and physical density, the titanium implant had a larger diameter than the intracanal post. Thus, irrespective of the lower physical density of the titanium implant, its larger diameter probably increased the artifacts [38]. Similarly, a previous study showed that irrespective of the application of the MAR algorithm in Picasso Trio and ProMax scanners, the Co-Cr alloy in the exomass resulted in a lower mean GV and higher SD than a titanium cylinder [17], which was in line with the present findings. Another study showed that the presence of three titanium dental implants in the exomass in the use of Cranex 3D, Orthophos SL-3D, and Z1 caused a significant reduction in the mean GV compared with the control status [36].

Aside from the type of metal object, the effect of the number of metal objects present in the exomass was also evaluated in this study. The results showed that the bilateral presence of metal objects in the exomass decreased the mean GV and increased the SD of GV compared with their unilateral placement. This finding indicates that higher-quality images can be expected in patients with fewer metal objects in the exomass. In other words, the number of metal objects in the exomass had an inverse correlation with the mean GV. Thus, the presence of a higher number of metal objects in the exomass results in overall image darkening, probably because of the dominance of hypodense over hyperdense artifacts, which are distributed nonhomogenously. Previous studies also demonstrated that a higher number of metal objects out of the FOV decreased the mean GV and increased noise [11, 28, 33].

The present results indicated that the SD of GV was minimum in the unilateral placement of titanium implants and maximum in the bilateral placement of Co-Cr intracanal posts and amalgam restorations, which was in line with a previous study [16]. Thus, based on the present results and the available literature, a higher number of metal objects with a high atomic number have a higher potential for image distortion [10]. Also, it has been demonstrated that the MAR algorithm is not successful in minimizing the effect of exomass-related artifacts on the GV [17]. Moreover, the MAR algorithm can have different effects on the mean GV depending on the source of artifact and the type of CBCT scanner [39]. Also, it increases the time required for image reconstruction and occupies a larger space for saving data [40]. Thus, its application is not recommended, and it was not used in the present study either.

In general, the present results indicate that in the case of using a small FOV for patients with metal objects of high density and atomic number in their oral cavity, the obtained CBCT images may have lower than expected quality. CBCT scans with a larger FOV probably have less noise due to the decreased exomass effect [11, 27, 28, 35]. However, they increase the patient's radiation dose, which should be taken into account.

When requesting CBCT, the clinician should ideally detect the possible sources of artifacts and their location relative to the ROI. Accordingly, the radiation dose can be adjusted to select a proper-size FOV and acquire an image with optimal quality.

This study had several strengths. Most previous studies on artifacts of metal objects located in the exomass were conducted on a polypropylene cylindrical phantom [5, 10, 17]. However, in the present study, metal objects were placed in a dry human mandible to better simulate in vivo conditions [38]. Also, ballistic gelatin was used to simulate the soft tissue. A recent study measured the mean and SD of GV and reported that ballistic gelatin was the best material to simulate soft tissue [19]. Nonetheless, generalization of results to the clinical setting should be done with caution because X-ray interferences are different in each patient [41]. Also, motion artifacts, which can be problematic in the clinical setting, were not present in this study. However, the current results can pave the way for further studies on other CBCT scanners and different dental materials in vitro with a better simulation of the clinical setting. Also, different voxel sizes and exposure parameters can be evaluated in future studies.

## 5. Conclusion

The use of small FOV increases the effect of exomass, which may negatively affect the image quality. Artifacts generated by metal objects in the exomass significantly decrease the GV of CBCT scans and adversely affect the image quality. Thus, the possible sources of artifacts and their location should be identified, and the radiation dose should be adjusted in order to use the proper-size FOV and preserve the image quality.

## Figures and Tables

**Table 1:** Descriptive findings regarding the mean GV based on the size of FOV and the type and number of metal objects (*n* = 10).

Group	Number	Minimum	Maximum	Mean	Std. error	Std. deviation	Variance
6 *∗* 6-Co-Cr-2	10.00	748.87	775.59	767.96	2.83	8.95	80.10
8 *∗* 8-Co-Cr-2	10.00	610.40	828.70	716.14	22.48	71.10	5055.31
6 *∗* 6-amalgam-2	10.00	451.50	609.20	545.50	17.88	56.53	3195.64
8 *∗* 8-amalgam-2	10.00	589.40	657.50	629.40	7.40	23.40	547.54
6 *∗* 6-amalgam-1	10.00	470.60	653.80	569.82	21.11	66.75	4455.92
8 *∗* 8-amalgam-1	10.00	358.30	689.20	502.04	31.63	100.02	10004.02
6 *∗* 6-titanium-2	10.00	841.70	848.27	845.65	0.59	1.87	3.50
8 *∗* 8-titanium-2	10.00	954.90	971.60	962.78	1.80	5.68	32.31
6 *∗* 6-titanium-1	10.00	705.80	790.50	763.38	9.10	28.78	828.35
8 *∗* 8-titanium-1	10.00	933.50	940.30	937.42	0.70	2.21	4.88
6 *∗* 6-control-1	10.00	884.80	892.30	889.20	0.82	2.60	6.75
6 *∗* 6-control-2	10.00	888.70	892.10	890.76	0.35	1.11	1.23
8 *∗* 8-control-2	10.00	951.80	960.90	955.38	0.77	2.44	5.94
8 *∗* 8-control-1	10.00	968.30	974.00	971.99	0.57	1.80	3.24
6 *∗* 6-Co-Cr-1	10.00	860.10	864.30	862.10	0.41	1.31	1.71
8 *∗* 8-Co-Cr-1	10.00	915.50	919.00	917.52	0.30	0.95	0.91

**Table 2:** Descriptive findings regarding the SD of GV based on the size of FOV and the type and number of metal objects.

Group	Number	Minimum	Maximum	Mean	Std. error	Std. deviation	Variance
6 *∗* 6-Co-Cr-2	10.00	50.60	60.90	54.84	1.07	3.38	11.41
8 *∗* 8-Co-Cr-2	10.00	69.90	98.70	82.15	2.82	8.91	79.39
6 *∗* 6-amalgam-2	10.00	61.80	76.70	68.62	1.76	5.57	31.00
8 *∗* 8-amalgam-2	10.00	60.50	70.30	63.43	0.98	3.09	9.52
6 *∗* 6-amalgam-1	10.00	69.70	88.10	77.27	1.79	5.65	31.97
8 *∗* 8-amalgam-1	10.00	76.00	99.10	84.04	2.14	6.77	45.77
6 *∗* 6-titanium-2	10.00	51.90	63.60	58.57	1.25	3.94	15.50
8 *∗* 8-titanium-2	10.00	46.40	59.60	51.78	1.51	4.78	22.86
6 *∗* 6-titanium-1	10.00	44.60	52.90	47.72	0.96	3.05	9.29
8 *∗* 8-titanium-1	10.00	39.80	53.80	44.18	1.42	4.49	20.17
6 *∗* 6-control-1	10.00	38.70	51.50	45.11	1.47	4.66	21.74
6 *∗* 6-control-2	10.00	42.70	55.30	47.32	1.25	3.96	15.67
8 *∗* 8-control-2	10.00	44.80	70.40	62.06	2.60	8.21	67.40
8 *∗* 8-control-1	10.00	42.80	55.50	48.69	1.34	4.22	17.85
6 *∗* 6-Co-Cr-1	10.00	56.00	61.80	59.75	0.58	1.84	3.39
8 *∗* 8-Co-Cr-1	10.00	44.50	53.70	48.40	0.93	2.93	8.59

## Data Availability

The datasets used and/or analyzed during the current study are available from the corresponding author on reasonable request.
